# Antimicrobial Photodynamic Therapy in the Treatment of Diabetic Foot Ulcers: A Pilot Randomized Clinical Trial

**DOI:** 10.1002/jbio.70278

**Published:** 2026-05-11

**Authors:** Maria Girlane Sousa Albuquerque Brandão, Mayra Gonçalves Menegueti, Roberto Bueno Filho, Luciano Rocha Mendonça, Ana Carolina Gomes, Idevania G. Costa, Soraia Assad Nasbine Rabeh

**Affiliations:** ^1^ Institute of Health Sciences University of International Integration of Afro‐Brazilian Lusophony – UNILAB Redenção Ceará Brazil; ^2^ Department of General and Specialized Nursing, School of Nursing University of São Paulo at Ribeirão Preto Ribeirão Preto São Paulo Brazil; ^3^ Hospital das Clínicas of the Faculty of Medicine of the University of São Paulo Ribeirão Preto São Paulo Brazil; ^4^ Portuguese Beneficence Hospital of Ribeirão Preto Ribeirão Preto São Paulo Brazil; ^5^ School of Nursing Lakehead University Thunder Bay Ontario Canada

**Keywords:** Bates‐Jansen, diabetic foot, oxidative stress, photodynamic therapy, wound care, wound healing

## Abstract

This pilot randomized clinical trial evaluated photodynamic therapy (PDT) in diabetic foot ulcers (DFUs), comparing an intervention group (IG, *n* = 5) with a control group (CG, *n* = 4). Participants were followed for 7 weeks. The IG received PDT, while the CG underwent conventional care. The microbiological profile was assessed by biopsy before and after treatment, and wound progression was evaluated using the Bates‐Jensen Wound Assessment Tool. In the IG, only one participant showed an increase in the number of bacterial species, while the others maintained the count with changes in species composition. In the CG, two participants presented an increase in bacterial species. All participants in the IG showed a reduction in ulcer size, with an average decrease of more than 63%, whereas two participants in the CG exhibited an increase in lesion area. These results suggest clinically relevant differences, supporting PDT as a promising adjuvant strategy for antimicrobial stewardship in DFUs.

## Graphical abstract

This randomized pilot study evaluated photodynamic therapy as an adjunctive treatment for diabetic foot ulcers compared with conventional care. The findings demonstrated a greater reduction in lesion area in the intervention group and potential benefits in the microbiological profile of the wounds, suggesting the clinical relevance and therapeutic potential of PDT in the management of these lesions.

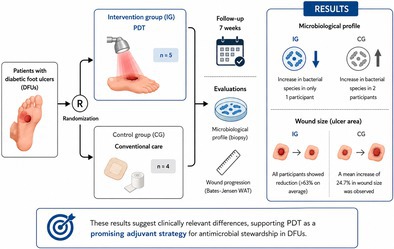

## 1 Introduction

Diabetic foot ulcers (DFUs) are among the most common complications of diabetes mellitus (DM). They are associated with high morbidity and mortality, as well as substantial financial costs due to hospitalization and amputations. This burden is further aggravated by the high incidence of infections, which significantly reduce the likehood of timely complete healing [1].

It is estimated that only 30%–40% of DFUs heal after 3 months of standard treatment. The longer a wound remains open, the more likely it is to enter chronic inflammatory cycles and develop bacterial biofilms. These factors contribute not only to delay in healing but also to the frequent and unnecessary use of antibiotics even in the absence of systemic infection and resistance. Such practices increase the risk of antimicrobial resistance. Approximately 20% of infected DFUs result in amputations [2], which, in addition to physical disability, often leads to serious emotional distress and mental health challenges for patients and their families [3].

Given the profound physical, emotional, and psychological consequences experienced by individuals with DFUs, optimizing wound healing is crucial. Effective wound management not only reduces the risk of infections, hospitalizations, and costs but also plays an important role in antimicrobial stewardship (AMS), by minimizing unnecessary antibiotic use and reducing the development of antimicrobial resistance [4]. Incorporating AMS principles into DFU care ensures that antibiotics are used judiciously and only when clinically indicated, while alternative strategies are employed to address local infection and promote healing. Therefore, exploring adjuvant technologies that complement conventional treatment is essential to improving outcomes reducing reliance on systemic antibiotics, and supporting stewardship efforts [5].

Among non‐antibiotic adjuvant alternatives that support antimicrobial stewardship, photodynamic therapy (PDT) stands out, combining laser light, oxygen, and a photosensitizer to induce antimicrobial effects [6, 7, 8]. The mechanism involves the generation of reactive oxygen species (ROS) through the interaction of the photosensitizer with light and oxygen, creating an environment highly toxic to bacterial colonies [9]. This technique can contribute to more favorable clinical outcomes in individuals with DFUs. Evidence suggests that combining conventional treatment with PDT is more effective in managing DFUs than conventional treatment alone [7, 10]. In Italy, one analysis reported that PDT reduced by 50% the time required to achieve outpatient healing in patients with DFUs, with a positive budgetary impact [11].

Although PDT has shown promising results, its efficacy in DFUs still requires clinical validation to fully understand its benefits and limitations, particularly as an adjuvant therapy. This underscores the importance of pilot studies capable of defining detailed protocols and assessing the feasibility and effectiveness of PDT in real‐world clinical settings. Another challenge is the estimation of sample sizes for randomized clinical trials (RCTs) on PDT in DFUs, as commonly used formulas require the definition of a clinical difference to calculate sample size. In this regard, the clinical differences in healing observed between intervention and control groups in this study provide a foundation for future sample size calculations and methodological design of more robust trials.

Thus, the objective of this study was to compare the microbiological profile of bacteria and the clinical evolution of ulcers in patients undergoing a PDT protocol in an intervention group compared with a control group.

## 2 Methods

### 2.1 Study Design

This is a pilot randomized clinical trial conducted in the outpatient services of two tertiary hospitals located in a city in the state of São Paulo, Brazil. The study was carried out between April 2023 and January 2024. Througout this section we describe a brief overview of the methods, as the great detail of our study protocol, including the Standard Operating Procedures (SOPs), has been published in the *WCET Journal* [12].

### 2.2 Study Population and Eligibility Criteria

In this study, convenience sampling was chosen, a non‐probabilistic approach, due to the difficulties encountered in recruiting participants. This strategy has been widely employed in clinical studies involving specific populations and innovative treatments such as PDT. For instance, a previous study with the same population and therapy also used convenience sampling to ensure feasibility without compromising methodological rigor or study objectives [13].

Inclusion criteria encompassed both sexes, age over 18 years, presence of a DFU, agreement to undergo two biopsies for microbiological profile evaluation, and availability to attend weekly outpatient visits for 7 weeks. Exclusion criteria included diagnosis or current treatment of malignancies, chronic kidney disease, or peripheral vascular insufficiency; suspected or confirmed osteomyelitis; ulcer area greater than 5 × 5 cm; ankle‐brachial index (ABI) below 0.7; and patients receiving other therapies.

### 2.3 Treatment Groups

#### 2.3.1 Intervention Group (IG)–PDT Application

In the IG, the DFUs were cleaned with saline solution (0.9%) and underwent instrumental debridement of necrosis or/and keratosis. Subsequently, methylene blue (1%) was applied to the wound using a 3 mL plastic pipette. The amount used varied according to the wound size (0.5 mL for wounds up to 4 cm^2^ and 1 mL for wounds larger than 4 cm^2^). After application, a five‐minute interval was timed [12].

Next, LASER light irradiation was performed (660 nm, 100 mW, 9 J, and 90 s per point). The technique involved point contact with a standardized distance of 1 cm between each point around the wound and 0.5 cm from the wound margin. After each participant, the device was disinfected with 70% liquid alcohol, and the tip was covered with transparent PVC plastic film [13].

**FIGURE 1:**
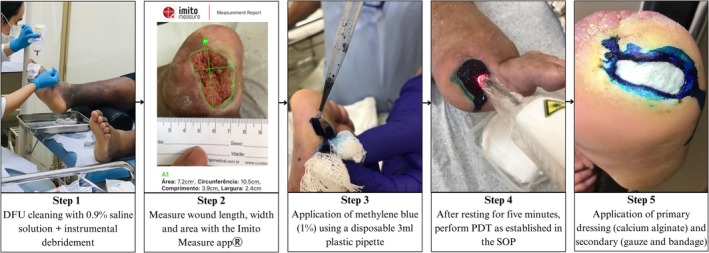
Illustration of the step‐by‐step sequence of the PDT protocol in the IG.

After PDT, a primary dressing of calcium alginate without silver was applied to cover the wound, protected with sterile gauze and crepe bandage. Each participant received a wound care kit with the prescribed dressing to change it at home between in‐person treatment with PDT. The dressing changes were carried out according to the level of exudation. Both the participant and the caregiver (when needed) were properly trained to perform dressing changes [12]. The summary of the PDT protocol is described in Figure 1.

#### 2.3.2 Control Group (CG)–Conventional Treatment Application

In the CG, ulcer cleansing was performed as previously described, following the same procedure as in the IG. The methylene blue was also used under the same conditions and concentration as in the IG to maintain the same wound bed coloration and, consequently, ensure blinding of participants and the research team. After applying methylene blue and the resting time, LASER light was irradiated using the same device and application techniques as in the IG. However, the device tip was blocked with a silicone rubber to prevent actual light irradiation on the wound [12].

After this procedure, a primary dressing of calcium alginate without silver was applied to cover the wound, protected with sterile gauze and crepe bandage. As in the IG, participants received the prescribed dressing to change at home, and both the participant and the caregiver (when needed) were properly trained to perform this procedure safely at home.

In both groups, treatment sessions were conducted at weekly intervals for six consecutive weeks by a nurse trained in laser therapy. All participants were instructed to return in the seventh week for outcome assessment. In accordance with the institutional protocol, patients who completed the 7 weeks were discharged from the study but continued receiving the standard treatment established by the outpatient clinic's professional team.

### 2.4 Assessments

#### 2.4.1 Epidemiological Data

The data collected included the following variables: (1) demographic information: age, sex, occupation, skin color, marital status, and education level; and (2) risk factor assessment: systemic diseases, duration of DM, hygiene, mobility, and medications. In addition, during the first week of treatment, glycated hemoglobin, Ankle‐Brachial Index, and the Classification System and Score in Comparing Outcome of Foot Ulcer Management (SINBAD) were evaluated [14].

#### 2.4.2 Microbiological Analysis

The reduction in the number of bacteria through tissue biopsy was selected as the primary outcome. For bacterial assessment, two biopsies were performed by a physician in week 1 and week 7. To carry out the procedure, sterile techniques were applied, using sterile material provided by the surgical center department, along with a sterile surgical drape and gown. The complete biopsy procedure was described in the SOP published by the *WCET Journal* [12]. None of the professionals from the microbiology department were aware of the participants' allocation.

#### 2.4.3 Clinical Evaluation of the Lesions

The clinical evolution of DFUs was always performed by the first author (M.G.S.A.B). Signs of wound improvement and healing rate was considered as secondary outcome. The lesions were assessed using the Bates‐Jensen Wound Assessment Tool (BWAT), culturally validated by Brazilian authors [15].

Application of the instrument was preceded by the analysis of wound length, width, and area, using the smartphone application *Imito Measure*. Photographic records were captured with an iPhone 7 camera, aided by a portable ring light. The measurements taken in the first and seventh weeks of follow‐up were considered for calculating the Ulcer Healing Index (UHI) [16].

### 2.5 Recruitment and Blinding

For recruitment, in both hospitals, patients were selected by the first author through outpatient visits. During consultations, researcher (M.G.S.A.B) assessed whether the patient met the eligibility criteria. If the person qualified, the researcher provided an invitation and explained the research process. Next, the biopsy procedure was performed by the physician, and the patient was randomized into one of the study groups using individual, sealed, and opaque envelopes [12].

In the present study, participants, physicians, research assistants, and the statistician were blinded to patient allocation. Therefore, only one researcher, responsible for applying PDT, was aware of the participants' allocation. In this case, it would not be technically possible for the researcher to remain blinded while using the light source and photosensitizer.

### 2.6 Statistical Analysis

The collected data were stored in Microsoft Excel spreadsheets and subsequently analyzed using SPSS, version 29. For descriptive data analysis, measures of central tendency (mean and median) and dispersion (range and standard deviation) were used for quantitative variables, while absolute and relative frequencies were used for categorical variables.

Inferential analysis of the reduction in wound area employed the nonparametric Mann–Whitney test, given the violation of the assumptions for the student's *t*‐test. Cohen's *d* index was also used to measure the effect size of the percentage change in DFU area at two time points (week 1 and week 7 of follow‐up). Statistical power analysis was performed using PASS (Power Analysis and Sample Size), version 15. This study adopted a significance level of *α* = 0.05.

### 2.7 Ethical Aspects

The research followed the principles of the Declaration of Helsinki and the ethical guidelines for studies involving human subjects, and it only began after approval by the Research Ethics Committees of both participating institutions. In addition, registration was obtained in the Brazilian Clinical Trials Registry (RBR‐2dm7t97).

Participation of individuals with DFU was entirely voluntary and formalized through signed informed consent in two copies. For anonymity purposes, participants were identified as P1, P2…P5, without reflecting the actual enrollment order in the study to prevent identification.

## 3 Results

**FIGURE 2:**
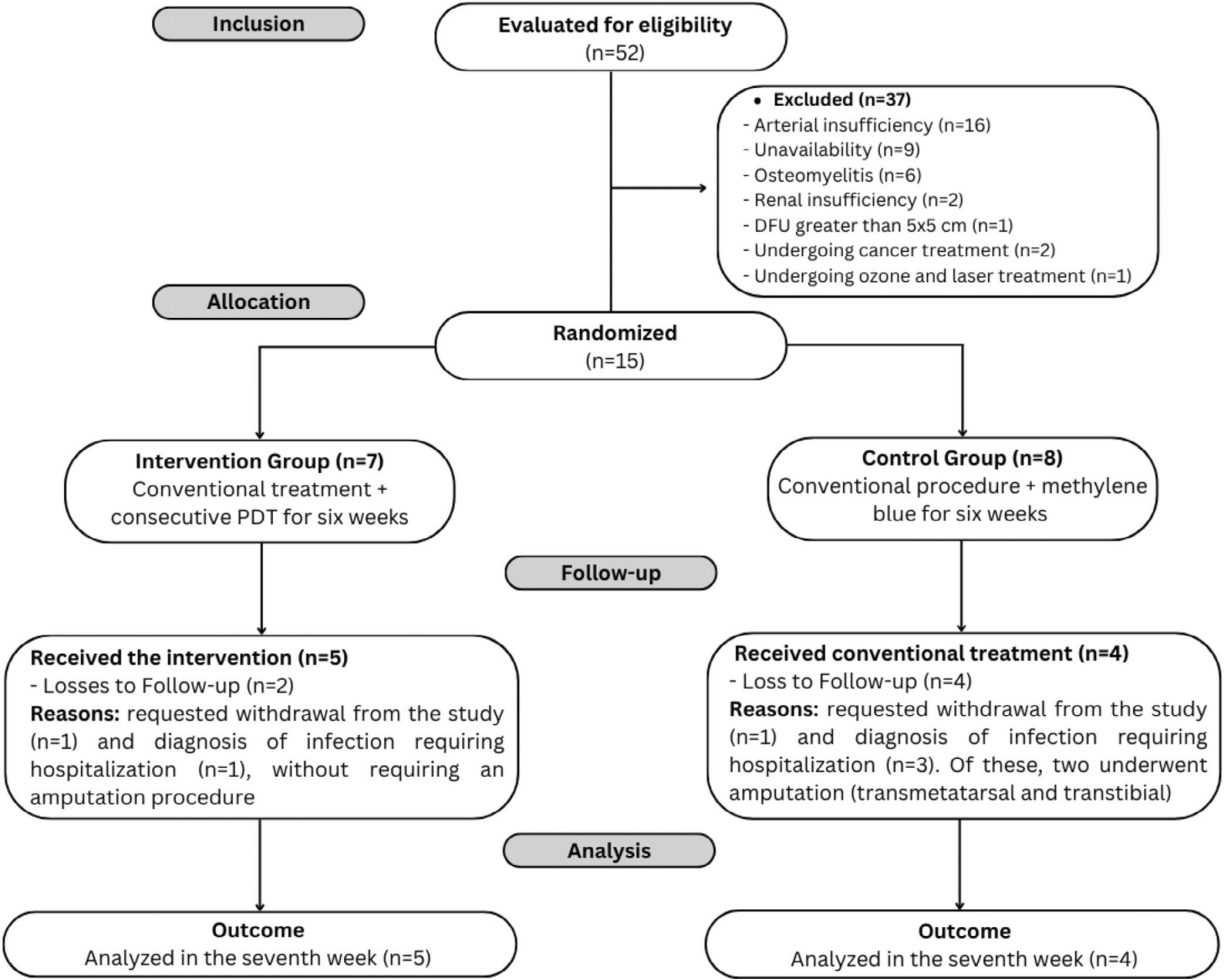
Flow diagram of participants at each stage of the study according to the CONSORT statement.

The study involved two groups of participants: IG (*n* = 5; 55.6%) and CG (*n* = 4; 44.4%). Figure 2 presents the follow‐up of participants in each phase of the study.

In the IG, most participants were female (*n* = 4; 80%), with a mean age of 54.40 years. The predominant education level in this group was high school (*n* = 3; 60%), and most were married or in a stable union (*n* = 4; 80%) and self‐identified as White (*n* = 5; 100%). In contrast, the CG showed an equal sex distribution (*n* = 2; 50% each), with a mean age of 60 years. This group presented greater educational diversity, with participants distributed across illiterate, incomplete elementary school, high school, and higher education levels (*n* = 1; 25% each category), and all self‐identified as Brown (*n* = 4; 100%) (Table 1).

**TABLE 1:** Distribution of sociodemographic characteristics of participants in the IG and CG.

Variables	Treatment groups			
	IG (*n* = 5)		CG (*n* = 4)	
	*N*	%	*N*	%
Sex				
Male	1	20.0%	2	50.0%
Female	4	80.0%	2	50.0%
Age (mean ± SD)	54.4 (±14.94)		60.0 (±17.02)	
Education level				
Illiterate	—	—	1	25.0%
Incomplete Elementary School	2	40.0%	1	25.0%
High School	3	60.0%	1	25.0%
Higher Education	—	—	1	25.0%
Occupation				
Employed	—	—	1	25.0%
Unemployed	3	60.0%	—	—
Retired/Pensioner	1	20.0%	2	50.0%
On sick leave/Disability benefit	1	20.0%	1	25.0%
Marital status				
Married/Stable union	4	80.0%	1	25.0%
Single	—	—	1	25.0%
Widowed	1	20.0%	1	25.0%
Divorced/Separated	—	—	1	25.0%
Race/Ethnicity				
White	5	100.0%	—	—
Brown	—	—	4	100.0%
Living arrangements				
Partner, children and/or others	3	60.0%	1	25.0%
Living only with partner	1	20.0%	—	—
Family members, without partner	—	—	2	50.0%
Living alone	1	20.0%	1	25.0%

All participants had a diagnosis of type 2 DM. The IG had a longer mean duration of diagnosis (27.6 years ±10.52) compared to the CG (14.75 years ±9.84). Regarding treatment, 60.0% (*n* = 3) of the IG used oral hypoglycemic agents, while 50.0% (*n* = 2) of the CG did; all IG participants (*n* = 5) used insulin, compared to 75.0% (*n* = 3) in the CG. Based on weight and height measurements, the mean BMI was 30.79 (±6.14) in the IG and 33.48 (±11.92) in the CG.

The values obtained for the Late Algorithm in the IG showed a mean of 3.2 (±1.8), ranging from 2 to 6. In the CG, the mean was 4 (±0), remaining constant within the range of 4 to 4. For the SINBAD score, the mean recorded was 2 (±0) in the IG. In the CG, the mean was 2 (±1), with values ranging from 1 to 3. Hypertension was present in 60% (*n* = 3) of the IG and 100% (*n* = 4) of the CG, and all participants were independent. Only one patient (allocated to the CG) was using adapted footwear at the time of data collection (Table 2).

**TABLE 2:** Distribution of clinical characteristics of participants in the IG and GC.

Variables	Treatment groups			
	IG (*n* = 5)		CG (*n* = 4)	
	*N*	%	*N*	%
Type of DM				
DM2	5	100.0%	4	100.0%
Duration of DM^a^ (years)	27.60 (±10.52)		14.75 (±9.84)	
Diabetes treatment				
Oral hypoglycemic agents	3	60.0%	2	50.0%
NPH and Regular insulin	5	100.0%	3	60.0%
Weight (kg)^a^	88.04 (±25.29)		95.15 (±46.05)	
Height (cm)^a^	1.68 (±0.09)		1.66 (±0.09)	
Body mass index^a^	30.79 (±6.14)		33.48 (±11.92)	
Glycated hemoglobin (HbA1c)^a^	7.26 (±0.40)		7.94 (±1.96)	
Ankle‐Brachial Index^b^	1.03 (±0.1)		1.01 (±0;10)	
Tardivo Algorithm	3.2 (±1.8)		4 (±0)	
Minimum and maximum	2–6		4–4	
SINBAD	2 (±0)		2 (±1)	
Minimum and maximum	2–2		1–3	
Associated diseases				
Arterial hypertension	3	60.0%	4	100.0%
Mobility				
Independent	5	100.0%	4	100.0%
Use of adapted footwear				
Yes	—	—	1	25.0%
No	5	100.0%	3	75.0%

Abbreviations: DM, diabetes mellitus; SINBAD, classification system and score in comparing outcome of foot ulcer management.

^a^ Mean and Standard Deviation.

^b^ ABI—member with the DFU.

In the microbiological profile assessment, it was observed that in the IG, only participant 3 showed an increase in the number of bacteria, from one to two bacterial species, with the addition of *Escherichia coli* alongside *Staphylococcus aureus* . In the CG, two participants (1 and 2) showed an increase in the number of bacterial species between biopsies, with *Staphylococcus aureus* and *Proteus mirabilis* identified in the second biopsies, compared to *Enterococcus gallinarum* and *Staphylococcus aureus* in the first biopsies, respectively. The remaining participants in both groups maintained the number of bacterial types throughout the study, although variations in species were observed between biopsies (Table 3).

**TABLE 3:** Microbiological profile of GI and GC participants, based on the results of the 1st and 2nd biopsies.

Treatment groups	Microbiological profile of diabetic foot ulcers			
	1st biopsy (1st week)		2nd biopsy (7th week)	
	Number of bacterial types	Bacterial species	Number of bacterial types	Bacterial species
Intervention group				
P1	1	*Staphylococcus aureus*	1	*Staphylococcus aureus*
P2	1	*Proteus mirabilis*	1	*Klebsiella pneumoniae*
P3	1	*Staphylococcus aureus*	2	*Staphylococcus aureus* and *Escherichia coli*
P4	2	*Staphylococcus aureus* and *Morganella morganii*	2	*Staphylococcus aureus and Morganella morganii*
P5	2	*Staphylococcus aureus* and *Morganella morganii*	2	*Staphylococcus aureus* and *Proteus mirabilis*
Control group				
P1	1	*Enterococcus galinarum*	2	*Staphylococcus aureus* and *Proteus mirabilis*
P2	1	*Staphylococcus aureus*	2	*Staphylococcus aureus* and *Proteus mirabilis*
P3	1	*Escherichia coli*	1	*Escherichia coli*
P4	1	*Morganella morganii*	1	*Pseudomonas aeruginosa*

The data in Table 4 show the variability in microorganism susceptibility profiles to different antibiotics in samples collected from the first and second biopsies of each group. Some antibiotics were tested only in specific biopsies, resulting in the indication “NT” (not tested) on several occasions.

**TABLE 4:** Antibiotic susceptibility profile of microorganisms found in ulcer biopsy samples from the IG and CG.

Antibiotics	Intervention group														
	1st biopsy							2nd biopsy							
	P1	P2	P3	P4		P5		P1	P2	P3		P4		P5	
	*S. aureus*	*P. mirabilis*	*S. aureus*	*S. aureus*	*M. morganii*	*S. aureus*	*M. morganii*	*S. aureus*	*K. pneumoniae*	*S. aureus*	*E. coli*	*S. aureus*	*M. morganii*	*S. aureus*	*P. mirabilis*
Amikacin	NT	S	NT	NT	S	NT	NT	NT	S	NT	S	NT	NT	NT	NT
Ampicillin	NT	S	NT	NT	R	NT	NT	NT	R	NT	NT	NT	NT	NT	NT
Amoxicillin	NT	NT	NT	NT	NT	NT	R	NT	NT	NT	R	NT	R	NT	R
Aztreonam	NT	S	NT	NT	NT	NT	S	NT	S	NT	NT	NT	S	NT	NT
Benzylpenicillin	NT	NT	NT	R	NT	R	NT	NT	NT	R	NT	R	NT	R	NT
Cefepime	NT	S	NT	NT	S	NT	S	NT	S	NT	S	NT	S	NT	S
Ceftazidime	NT	S	NT	NT	S	NT	R	NT	S	NT	S	NT	S	NT	S
Cefotaxime	NT	S	NT	NT	NT	NT	NT	NT	S	NT	NT	NT	NT	NT	NT
Cefoxitin	R	NT	NT	NT	NT	NT	NT	R	NT	NT	NT	NT	NT	NT	NT
Cefazoline	NT	NT	S	S	NT	S	NT	NT	NT	S	NT	S	NT	NT	NT
Clindamycin	S	NT	R	S	NT	R	NT	I	NT	R	NT	S	NT	NT	NT
Ceftriaxone	NT	NT	NT	NT	S	NT	R	NT	NT	NT	S	NT	R	NT	R
Cefuroxime	NT	NT	NT	NT	NT	NT	NT	NT	NT	NT	I	NT	NT	NT	R
Ciprofloxacin	I	S	NT	NT	R	NT	S	I	S	NT	R	NT	R	NT	S
Chloramphenicol	NT	NT	NT	NT	NT	NT	NT	NT	S	NT	NT	NT	NT	NT	NT
Colistin	NT	R	NT	NT	NT	NT	NT	NT	R	NT	NT	NT	NT	NT	NT
Daptomycin	S	NT	S	S	NT	NT	NT	S	NT	NT	NT	S	NT	NT	NT
Erythromycin	R	NT	NT	NT	NT	NT	NT	R	NT	NT	NT	NT	NT	NT	NT
Ertapenem	NT	S	NT	NT	S	NT	S	NT	S	NT	S	NT	S	NT	S
Gentamicin	S	S	S	S	R	S	S	S	S	S	R	S	R	I	S
Imipenem	NT	NT	NT	NT	I	NT	NT	NT	S	NT	NT	NT	NT	NT	NT
Levofloxacin	I	S	R	R	NT	I	NT	I	S	R	NT	I	NT	I	NT
Linezolid	S	NT	S	S	NT	S	NT	S	NT	S	NT	S	NT	S	NT
Meropenem	NT	S	NT	NT	S	NT	S	NT	S	NT	S	NT	S	NT	S
Oxacillin	R	NT	S	R	NT	R	NT	R	NT	R	NT	R	NT	R	NT
Penicillin G	R	NT	NT	NT	NT	NT	NT	R	NT	NT	NT	NT	NT	NT	NT
Piperacillin	NT	S	NT	NT	S	NT	S	NT	S	NT	S	NT	S	NT	S
Rifampicin	S	NT	R	S	NT	S	NT	S	NT	R	NT	S	NT	R	NT
Sulbactam	NT	NT	NT	NT	NT	NT	NT	NT	S	NT	NT	NT	NT	NT	NT
Teicoplanin	S	NT	S	S	NT	S	NT	S	S	NT	NT	S	NT	NT	NT
Tigecycline	NT	NT	S	S	NT	S	NT	NT	NT	S	S	S	NT	S	NT
Tobramycin	NT	S	NT	NT	NT	NT	NT	NT	S	NT	NT	NT	NT	NT	NT
Trimethoprim	S	S	S	S	NT	S	NT	S	S	S	NT	NT	NT	S	NT
Vancomycin	S	NT	S	S	NT	S	NT	S	S	S	NT	S	NT	S	NT

Antibiotics	Control group									
	1st biopsy				2nd biopsy					
	P1	P2	P3	P4	P1		P2		P3	P4
	*E. galinarum*	*S. aureus*	*E. coli*	*M. morganii*	*S. aureus*	*P. mirabilis*	*S. aureus*	*P. mirabilis*	*E. coli*	*P. aeruginosa*
Amikacin	NT	NT	S	S	NT	NT	NT	NT	S	S
Ampicillin	S	NT	R	R	NT	NT	NT	NT	R	NT
Amoxicillin	NT	NT	NT	R	NT	S	NT	NT	NT	NT
Aztreonam	NT	NT	S	S	NT	S	NT	S	S	I
Benzylpenicillin	NT	NT	S	NT	R	NT	NT	NT	S	NT
Cefepime	NT	NT	S	S	NT	S	NT	NT	S	I
Ceftazidime	NT	NT	S	NT	NT	S	NT	S	S	NT
Cefotaxime	NT	NT	S	NT	NT	NT	NT	S	S	NT
Cefoxitin	NT	NT	NT	NT	NT	NT	NT	NT	NT	NT
Cefazoline	NT	NT	NT	NT	S	NT	NT	NT	NT	NT
Clindamycin	NT	S	NT	NT	S	NT	R	NT	NT	NT
Ceftriaxone	NT	NT	NT	NT	NT	S	NT	S	NT	NT
Cefuroxime	NT	NT	S	NT	NT	I	NT	NT	S	NT
Ciprofloxacin	NT	NT	R	R	NT	S	NT	R	R	I
Chloramphenicol	NT	NT	S	NT	NT	NT	NT	NT	S	NT
Colistin	NT	NT	S	R	NT	NT	NT	NT	S	S
Daptomycin	NT	NT	NT	NT	S	NT	NT	NT	NT	NT
Erythromycin	NT	NT	NT	NT	NT	NT	NT	NT	NT	NT
Ertapenem	NT	NT	S	S	NT	S	NT	NT	S	NT
Gentamicin	NT	S	S	R	S	NT	NT	R	S	NT
Imipenem	NT	NT	S	I	NT	NT	NT	NT	S	I
Levofloxacin	NT	I	R	R	I	NT	I	NT	R	I
Linezolid	S	S	NT	NT	S	NT	S	NT	NT	NT
Meropenem	NT	NT	S	S	NT	S	R	S	S	S
Oxacillin	NT	S	NT	NT	S	R	NT	NT	NT	NT
Penicillin G	NT	R	NT	NT	NT	NT	NT	NT	NT	NT
Piperacillin	NT	NT	R	S	NT	S	NT	S	R	I
Rifampicin	NT	S	NT	NT	S	R	NT	NT	NT	NT
Sulbactam	NT	NT	NT	NT	NT	NT	NT	NT	NT	NT
Teicoplanin	S	S	NT	NT	S	NT	S	NT	NT	NT
Tigecycline	NT	S	R	I	S	NT	NT	NT	R	NT
Tobramycin	NT	NT	S	R	NT	NT	NT	NT	S	S
Trimethoprim	NT	S	S	R	S	NT	R	NT	S	NT
Vancomycin	R	S	NT	NT	S	NT	S	NT	NT	NT

Abbreviations:

**Table:** 

S	Sensitive	I	Intermediate	R	Resistant	NT	Not tested

In the IG, the main microorganisms identified were *Staphylococcus aureus* , *Proteus mirabilis* , and *Morganella morganii* . The antibiotics showing the highest susceptibility included vancomycin, teicoplanin, linezolid, meropenem, and trimethoprim, with a predominance of “sensitive” (S) results in several samples tested. In contrast, resistance was more frequent for ampicillin, rifampicin, and oxacillin.

In the CG, prominent microorganisms included *Escherichia coli* , *Staphylococcus aureus* , and *Proteus mirabilis* . Susceptibility was most evident for antibiotics such as vancomycin, meropenem, gentamicin, teicoplanin, and aztreonam. Resistance was more frequent for ampicillin, levofloxacin, rifampicin, and colistin. No pattern of improvement was observed in either group, meaning there were no cases in which a microorganism initially resistant became sensitive after 7 weeks.

Table 5 shows the Ulcer Healing Index (UHI). In the IG, all participants had UHI values > 0, indicating a reduction in DFU size. The UHI ranged from 0.46 (P4) to 0.92 (P5), suggesting positive progression in the healing process over the analyzed period. In contrast, in the CG, two patients had UHI > 0, but one of them (P3) had a value very close to 0, indicating a minimal reduction in wound size, while two patients had UHI < 0 due to an increase in wound size.

**TABLE 5:** Ulcer Healing Index of participants in the IG and CG.

Variable	Intervention group					Control group			
	P1	P2	P3	P4	P5	P1	P2	P3	P4
Ulcer Healing Index	0.60	0.61	0.56	0.46	0.92	−1.12	0.65	0.19	−0.68

*Note:* UHI = 1—Means total re‐epithelialization; UHI = 0—Indicates signs of re‐epithelialization; UHI > 0—Means reduction in the lesion area; UHI < 0—Means increase in the lesion area.

Table 6 shows the evolution of 13 subitems of the Bates‐Jensen Wound Assessment Scale, comparing baseline values (week 1) and final values (week 7) for the intervention and control groups. The scale ranges from 5 to 1, with 5 being the worst score and 1 the best. In the IG, all participants showed a reduction in scale values over the weeks, indicating significant improvements in wound conditions. Subitems such as necrotic tissue type, exudate amount, and epithelialization stood out for showing consistent progress toward the best scale values.

**TABLE 6:** Comparison of values obtained for each of the 13 subitems of the Bates‐Jensen scale at weeks 1 and 7 in the IG and CG.

Variable	Intervention group					Control group			
	P1	P2	P3	P4	P5	P1	P2	P3	P4
	1st–7th	1st–7th	1st–7th	1st–7th	1st–7th	1st–7th	1st–7th	1st–7th	1st–7th
1. Size	2–1	1–1	1–1	1–1	1–1	1–2	1–1	1–1	1–1
2. Depth	2–2	2–2	2–2	2–2	2–2	2–2	2–2	2–2	3–3
3. Edges	2–2	4–3	3–2	3–2	2–2	3–3	4–4	3–2	3–3
4. Undermining	2–1	2–2	2–1	2–1	1–1	1–1	2–3	2–1	5–5
5. Necrotic tissue type	2–1	1–1	2–1	1–1	2–1	1–2	3–1	2–1	2–2
6. Necrotic tissue amount	4–1	1–1	2–1	1–1	2–1	1–3	2–1	3–1	4–3
7. Exudate type	4–3	4–2	3–2	4–1	3–1	4–4	3–3	4–4	4–4
8. Exudate amount	4–3	4–2	3–2	3–1	3–1	4–4	4–4	4–3	5–4
9. Skin color surrounding wound	2–1	3–2	3–3	3–1	3–3	3–3	3–3	1–1	2–2
10. Peripheral tissue edema	2–1	1–1	1–1	1–1	1–1	1–1	1–1	1–1	1–1
11. Peripheral tissue induration	1–1	1–1	1–1	1–1	1–1	1–1	1–1	1–1	1–1
12. Granulation tissue	4–2	4–2	4–2	4–2	2–2	4–4	4–2	4–2	4–3
13. Epithelialization	5–3	5–4	4–4	4–2	4–2	5–5	5–5	5–2	5–4
Total score	36–22	33–24	31–24	30–18	27–19	31–34	35–31	33–22	40–36
Change in scale value	Reduction	Reduction	Reduction	Reduction	Reduction	Increase	Reduction	Reduction	Reduction

In the CG, although some improvements were observed, such as in necrotic tissue type and epithelialization, the progression was less uniform. One participant showed an increase in the total score between week 1 and week 7, contrasting with the pattern observed in the IG.

The Table 7 compares the baseline and final wound area measurements (in cm^2^) between the IG and CG. In the IG, the mean area decreased from 2.76 to 0.96 cm^2^, with a consistent reduction in the median (2.10 to 0.70 cm^2^) and in the minimum and maximum values (1.30–6.10 to 0.20–2.40 cm^2^). In the CG, there was an increase in the mean area from 3.02 to 3.30 cm^2^, with greater variation in the standard deviation (0.84 to 1.57) and an increase in maximum values (3.80 to 5.10 cm^2^).

**TABLE 7:** Comparison of baseline and final wound area measurements (cm^2^).

Variables	Intervention group		Control group	
	Initial	Final	Initial	Final
Media	2.76	0.96	3.03	3.30
Median	2.10	0.70	3.05	3.40
Standard Deviation	1.94	0.84	0.84	1.57
Minimum	1.30	0.20	2.20	1.30
Maximum	6.10	2.40	3.80	5.10

Although this difference is clinically relevant, as expected, the inferential analysis comparing the mean reduction in these areas did not reveal a statistically significant difference between groups, using the nonparametric Mann–Whitney test with exact *U* distribution (*p* = 0.19, two‐tailed test). Indeed, with nine participants, a post hoc statistical power analysis revealed, considering a significance level of *α* = 0.05, an achieved power of 32.3%.

The effect size analysis, using Cohen's *d* coefficient, revealed a clinically important difference between groups. In the IG, the mean percentage reduction in wound area was 63.5%, whereas in the CG, a mean increase of 24.7% in wound size was observed, indicating a large effect size (*d* = 1.44, with Hedges' correction).

## 4 Discussion

In the present study, the duration of DM was relatively long, as were hemoglobin levels and BMI. Moreover, only one participant used adapted footwear. Several studies corroborate that these factors can impair the healing process [17, 18, 19]. Nevertheless, these patients reflect a real‐world scenario characterized by multiple complications.

Another problem frequently observed in people with DFU is the high occurrence of infections, often associated with the presence of resistant microorganisms [20, 21]. Antimicrobial resistance poses a significant global threat to public health, primarily driven by the overuse of these medications, which has consequences for both individual patients and the community, as it facilitates the spread of resistant pathogens among individuals [22]. In this context, adjuvant therapies such as PDT become relevant, as they can assist in microbial control without promoting the development of resistance, thus contributing to antimicrobial stewardship actions.

Previous investigations indicate that PDT plays an important role in controlling infection‐causing bacteria and in decreasing antimicrobial resistance; therefore contributing to AMS [8, 23]. Accordingly, this study aimed to evaluate the efficacy of PDT in a real‐world setting. Initially, fewer losses due to infection were observed in the IG; only one participant developed an infection.

It should be emphasized that the presence of microorganisms is expected in DFUs, as these lesions are naturally hosts a community of microorganisms. They are exposed to commensal skin bacteria, which can colonize the wound and form microbial communities often protected by biofilm. Colonization in this context is defined as the presence of multiple types of bacteria without eliciting an evident immune response in the host. However, in many cases of DFU, worsening occurs, characterizing an infection that establishes when bacteria overcome host defenses [24]. This situation can lead to overuse of systemic antibiotics, which is often driven by diagnostic uncertainty and limited non‐antibiotic options, enabling microbes to adapt and develop resistance [25, 26]. Therefore, safe infection control strategies, including the use of evidence‐based antimicrobial dressings, such as methylene blue associated with LASER, are essential to reduce this risk and advance antimicrobial stewardship nationally and worldwide.

In this regard, bacterial control was defined as the primary outcome, focusing on the number of bacterial types. Biopsy evaluations showed that in the IG, only one participant exhibited an increase in the number of bacterial species, while the others maintained the same number of bacterial types throughout the study, although changes in the identified species were observed. These findings suggest that PDT has the potential to contribute to limiting bacterial colonization, consistent with studies highlighting its antimicrobial action [9, 27].

In the CG, however, two participants showed an increase in the number of bacterial species, including complex combinations such as *Staphylococcus aureus* and *Proteus mirabilis* . It is pertinent to note that changes in bacterial species may be related to various environmental factors and treatments. Continuous exposure to hospital or home environments, which may contain contaminated surfaces and materials, is a potential source of new contamination. Additionally, during bathing, inadequate wound protection may lead to the acquisition of different bacterial species.

A study that administered six PDT sessions to 55 patients in two countries (Germany and Italy), three times per week, showed a significant reduction in bacterial load after a single treatment (*p* < 0.001) [28]. This reduction in Colony Forming Units of bacteria counts after methylene blue associated with LASER appears to be related to decreased microbial viability. Authors report that patients treated with PDT may experience up to a 17% reduction in microbial viability, creating a more favorable environment for healing and less need for systemic antibiotics [29].

The most common bacteria in the microbiological profile analysis were *Staphylococcus aureus* , *Proteus mirabilis* , and *Morganella morganii* , resistant to classical antibiotics such as clindamycin, ceftriaxone, amoxicillin, and ciprofloxacin. Brazilian studies analyzing the microbiological profile of DFUs via biopsy corroborate the recurrence of these bacteria, commonly resistant, particularly to clindamycin and ciprofloxacin [20, 21].

This finding reflects the increasing bacterial resistance in people with DM, especially to antibiotics commonly used to treat DFU infections [21]. These outcomes demonstrate a safe, non‐cytotoxic strategy that reduces antibiotic use and supports antimicrobial stewardship. They also underscore the need for caution against the repeated or prolonged use of antibiotics in individuals with diabetic foot ulcers (DFUs), as misuse contributes to antimicrobial resistance and may further impair renal function already compromised by diabetic microangiopathy [30].

These findings further support the adoption of PDT as an adjuvant therapy in DFU management. It is pertinent to emphasize that applying PDT soon after lesion onset would be ideal to promote early microbial control, minimize antibiotic use, and limit lesion progression [8, 10].

Regarding the secondary outcome of clinical wound evolution, significant clinical improvements in the healing process were observed. In the IG, the mean DFU area decreased from 2.76 to 0.96 cm^2^, whereas in the CG, the total DFU area increased. These results align with other clinical studies showing a greater reduction in DFU area in the PDT‐treated group [13, 31], demonstrating that although the microbiological profile remained relatively stable, PDT effectively promoted DFU healing.

Indeed, examining the UHI, all IG participants had UHI > 0, indicating a reduction in DFU size. In contrast, in the CG, two participants had UHI < 0 due to lesion area increase, corroborated by Cohen's *d*. This result aligns with Aureliano, who reported a superior UHI reduction in the PDT‐treated group. These findings reinforce that PDT can be employed as an adjuvant therapy for DFUs. Besides promoting positive effects on healing, it is a device‐mediated intervention that is easy to handle and transport, with favorable adherence among the target population, being considered painless and non‐invasive [7].

In addition to greater healing progress, the IG showed improvements in key Bates‐Jensen scale items, especially necrotic tissue type, exudate amount, and epithelialization. These positive changes underscore the beneficial impact of PDT on wound tissue quality. Consistent with this evidence, a cohort study with 22 DFU patients also reported significant improvements in these subitems [7].

During follow‐up, no adverse events were reported. None of the IG participants reported complaints or required amputation during the treatment. In the CG, two participants required amputation due to deep tissue infection. A Brazilian study showed that the amputation rate was 35 times lower in the PDT‐treated group (*n* = 18), with only one case, compared to amputations in all CG participants (*n* = 16) [31]. Brocco et al. [32] also reported fewer amputations in PDT‐treated DFU patients. These findings emphasize the importance of expanding adjuvant therapies such as PDT to reduce the risk of osteomyelitis and, consequently, lower the likelihood of amputation [10, 33, 34].

This study has some limitations that must be considered when interpreting the results. First, microbial load was not recorded, limiting microbiological analyses to identification of microorganisms before and after treatment. Second, the small sample size, due to low patient turnover, difficulties in identifying patients within the healthcare network, and a high number of exclusions (*n* = 37), including individuals with circulatory insufficiency and patients facing logistical barriers due to living far from the study site.

For future studies, it is recommended to apply this protocol with quantitative microbial load analysis in a clinical trial conducted in real‐world settings across different geographic regions. Additionally, the inclusion of imaging exams, such as Doppler, is recommended to more sensitively assess blood flow, allowing the identification of patients with minor obstructions and expanding the pool of candidates for PDT.

## 5 Conclusion

The study's findings reinforce the potential of PDT as a promising adjuvant intervention in the treatment of DFUs and for advancing AMS. The group receiving PDT demonstrated superior outcomes in the Ulcer Healing Index, reductions in wound area, and improvements on the Bates‐Jensen scale, with no reported adverse events or amputations during follow‐up.

These results highlight the clinical relevance of PDT in promoting wound healing and reducing the risks of infection and amputation, particularly in the context of rising bacterial resistance. While PDT demonstrated consistent and encouraging results in accelerating healing, it should be integrated within a comprehensive treatment plan.

Optimal glycemic control, reduction of plantar pressure through appropriate footwear, and daily foot care remain essential for complete tissue repair; however, these measures alone are not sufficient to achieve the desired outcome. PDT should therefore be considered as an additional component of a multifaceted approach to managing such complex wounds.

With a standardized protocol now available, healthcare managers and professionals may consider implementing this therapy to expand treatment options for individuals with DFUs, especially those experiencing difficulties in wound healing.

## Author Contributions

**Maria Girlane Sousa Albuquerque Brandão:** methodology, data collection, writing – original draft, writing – review and editing. **Mayra Gonçalves Menegueti:** methodology, investigation, data collection, writing – original draft, writing – review and editing. **Roberto Bueno Filho:** data collection, writing – review and editing. **Luciano Rocha Mendonça:** data collection, writing – review and editing. **Ana Carolina Gomes:** data collection, writing – review and editing. **Idevania G. Costa:** methodology, writing – original draft, writing – review and editing. **Soraia Assad Nasbine Rabeh:** methodology, data collection, writing – original draft, writing – review and editing. All authors have read and agreed to the published version of the manuscript.

## Funding

The authors have nothing to report.

## Conflicts of Interest

The authors declare no conflicts of interest.

## Data Availability

The data that support the findings of this study are available from the corresponding author upon reasonable request.
